# Unconventional metabolites in chromatin regulation

**DOI:** 10.1042/BSR20211558

**Published:** 2022-01-20

**Authors:** Liubov Gapa, Huda Alfardus, Wolfgang Fischle

**Affiliations:** Biological and Environmental Science and Engineering Division, King Abdullah University of Science and Technology, Thuwal 23955, Saudi Arabia

**Keywords:** chromatin, DNA, epigenetcis, histones, metabolites, regulation

## Abstract

Chromatin, the complex of DNA and histone proteins, serves as a main integrator of cellular signals. Increasing evidence links cellular functional to chromatin state. Indeed, different metabolites are emerging as modulators of chromatin function and structure. Alterations in chromatin state are decisive for regulating all aspects of genome function and ultimately have the potential to produce phenotypic changes. Several metabolites such as acetyl-CoA, S-adenosylmethionine (SAM) or adenosine triphosphate (ATP) have now been well characterized as main substrates or cofactors of chromatin-modifying enzymes. However, there are other metabolites that can directly interact with chromatin influencing its state or that modulate the properties of chromatin regulatory factors. Also, there is a growing list of atypical enzymatic and nonenzymatic chromatin modifications that originate from different cellular pathways that have not been in the limelight of chromatin research. Here, we summarize different properties and functions of uncommon regulatory molecules originating from intermediate metabolism of lipids, carbohydrates and amino acids. Based on the various modes of action on chromatin and the plethora of putative, so far not described chromatin-regulating metabolites, we propose that there are more links between cellular functional state and chromatin regulation to be discovered. We hypothesize that these connections could provide interesting starting points for interfering with cellular epigenetic states at a molecular level.

## Introduction

Chromatin is a macromolecular complex composed of distinct molecules. The fundamental, repeating unit of chromatin, the nucleosome, is composed of 146 base pairs (bp) of double-stranded DNA wrapped around an octamer made up of two copies each of the core histones H2A, H2B, H3 and H4 [1]. Linear stretches of DNA packaged into nucleosomes are arranged into higher order structures by additional proteins including the linker histone H1, RNA and the involvement of other molecules to form chromatin.

The regulation of chromatin organization is crucial for all aspects of DNA biology including genome replication, gene expression, repair of DNA damage and meiotic recombination. Chromatin organization is directed by a plethora of chemical modifications on any of the different histone proteins or via methylation and its oxidation products of DNA. The modification reactions occur either nonenzymatically, or via the action of specialized chromatin-modifying enzymes (in context of histones, these are often referred to as writers for modifying enzymes and erasers for demodifying enzymes) [2]. Chromatin modifications may modulate histone–DNA complex stability and/or intranucleosome interactions. Also, specialized chromatin reader proteins can recognize the modifications and provoke downstream regulatory effects. Furthermore, chromatin remodeling enzymes organize nucleosome positioning, swap histone components for specific variants, or exchange nucleosomes at different positions. DNA accessibility and regulatory chromatin interactions are controlled at multiple scales including global effects such as the massive changes of chromosome compaction observed during the cell cycle, as well as locus-specific effects that direct the activity of individual genes [3].

The physiological state of a cell feeds into the regulation of chromatin not only via the supply of the building blocks of chromatin (i.e. nucleotides for DNA and amino acids for histone proteins) and energy status (i.e. driving chromatin remodeling processes which are dependent on adenosine triphosphate (ATP) hydrolysis) but also via a rapidly growing range of metabolites. The molecules that serve as substrates for enzymatic and nonenzymatic histone modifications and cofactors of chromatin-modifying enzymes have been of particular interest. Well-described metabolites in this context include nicotinamide adenine dinucleotide (NAD^+^), ATP, S-adenosylmethionine (SAM), α-ketoglutarate, acetyl-CoA and different acyl-CoA moieties. Their chromatin biology has been extensively reviewed in the recent literature [4–13]. In this article, we want to describe other less-frequently discussed metabolites that play various roles in chromatin regulation and assess whether there are additional modes of function besides the paradigm chromatin modification context.

## Molecular mechanisms of metabolites in regulating chromatin

**Figure 1:**
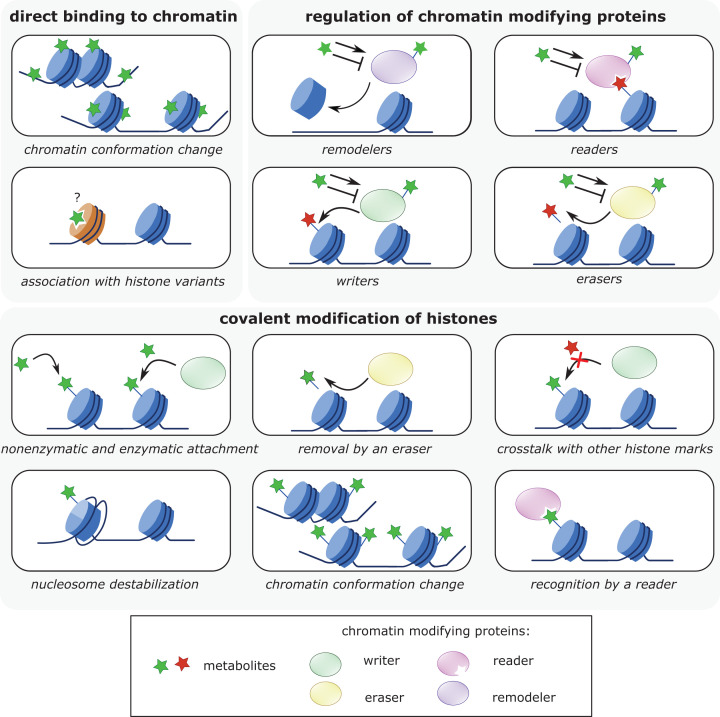
Molecular mechanisms of chromatin regulation by cellular metabolites Metabolites interact with chromatin and chromatin-modifying proteins via binding and covalent modification (gray boxes). Different modes of function are illustrated for each category of metabolite–chromatin interaction. The question mark signifies the unknown functional relevance of the interaction.

**Figure 2:**
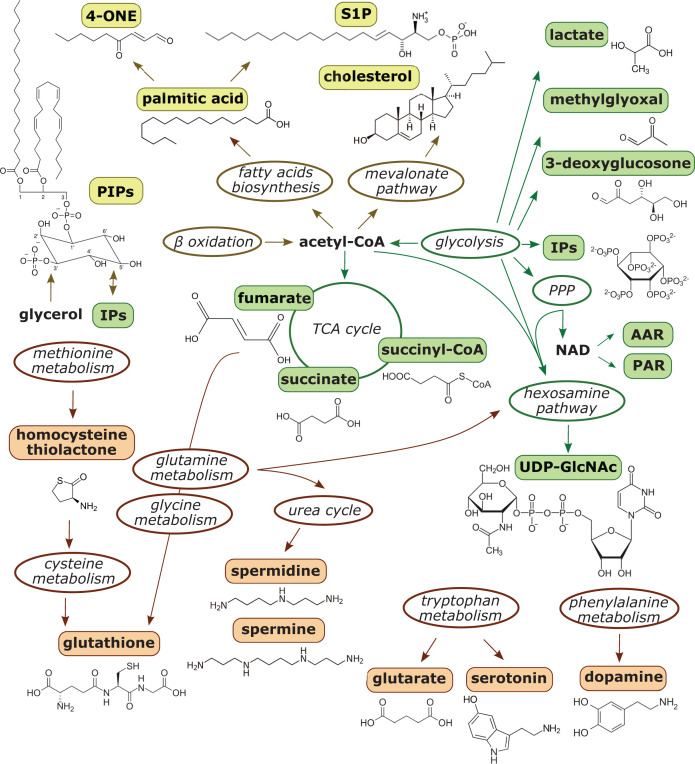
Origins of unconventional metabolites with potential for chromatin regulation Chromatin-regulating metabolites (highlighted with colored boxes) originate from many cellular processes—including carbohydrate (green), lipid (yellow), and amino acids (orange) metabolic pathways (encircled). The structural formula of the different metabolites discussed in this article are presented, with exception of AAR and PAR. Intermediate steps of biosynthetic or degradation pathways are omitted for clarity. Abbreviations: 4-ONE, 4-oxo-2-nonenal; AAR, acetyl-adenosine-diphosphate-ribose; IP, inositol polyphosphate; NAD, nicotinamide adenine dinucleotide; PAR, poly-adenosine-diphosphate-ribose; PIP, phosphatidylinositol phosphate (phosphoinositide); PPP, pentose phosphate pathway; S1P, sphingosine-1-phosphate; TCA, tricarboxylic acid; UDP-GlcNAc, uridine diphosphate N-acetylglucosamine.

Considering putative modes of metabolite function on chromatin, different mechanisms of action can be envisioned (Figure 1). Metabolites can modulate chromatin directly via interaction with DNA or histones, thereby affecting the stability of nucleosomes or internucleosomal interactions that are crucial for establishing higher order (i.e. decondensed or compacted) chromatin states. Metabolites interacting with chromatin can also interfere with chromatin modification or remodeling processes directly or via the induced chromatin states. Further, metabolites can serve as substrates for chromatin modifications. Besides being attached to chromatin via the action of specific writer enzymes, it is now well established that different metabolites can directly target histones for nonenzymatic covalent modification [2,14]. Histones accumulate stable nonenzymatic modifications because of their long half-lives and nucleophilic sites particularly enriched in their exposed N-terminal regions. In this context, it has been speculated that histones adopt the roles of ‘sponges’ in cells as part of an epigenetic feedback loop in metabolic adaptation [11]. In an indirect fashion, metabolites regulate the activity and function of chromatin-modifying writer and eraser enzymes or chromatin remodelers. Lastly, metabolites can control the binding activities of readers recognizing and functionally translating chromatin modifications. In the following, we will discuss examples of different less common or more unconventional metabolites originating from the carbohydrate, lipid and amino acid metabolic pathways that have less-recognized functions in chromatin regulation but that cover a spectrum of these different chromatin-regulatory mechanisms (Figure 2).

## Chromatin-regulating metabolites originating from carbohydrates

### Lactate

Lactate is a product of anaerobic pyruvate reduction and thereby originates from glycolysis. It serves as an energy source, precursor of gluconeogenesis, and a signaling molecule [15]. Lactate has several roles in epigenetic regulation. It promotes histone acetylation and gene expression in cell culture as an endogenous inhibitor of histone deacetylase (HDAC) activity [16], as well as via providing acetyl-CoA upon oxidation.

Recently, lactate was found covalently attached to different histone lysine residues. The modifications seem to be carried out by the p300 co-activator and enzyme that has been implied in different histone lysine acylation reactions [13]. Histone lactylation levels were shown to increase in a dose-dependent manner upon providing exogenous lactate, as well as after stimulation of endogenous production. Histone lactylation was found on 28 conserved sites in human and mouse cell lines, on all of the core histones H2A, H2B, H3, and H4 [17]. The modification is generally promoting transcription, possibly through mechanisms similar to other histone acylation post-translational modifications (PTMs) by disturbing internucleosomal contacts and decondensing chromatin [13]. Particularly, histone lactylation was connected to facilitating the expression of the YTHDF2 RNA-binding protein in ocular melanoma cells [18]. Another study demonstrated that in macrophages, increased lactate levels lead to histone lactylation on promoters of profibrotic genes in a model of lung fibrosis [19]. A connection of lactate and gene regulation was made with a transcription factor, Glis1 that was shown to specifically activate glycolytic genes, subsequently increasing acetyl-CoA and lactate levels in mouse embryonic fibroblasts (MEFs). This in turn led to increase in pluripotency loci expression via histone acetylation and lactylation mechanisms [20].

### Products of the tricarboxylic acid cycle

The best studied intermediate of the Krebs cycle in chromatin regulation is α-ketoglutarate that serves as a co-substrate of α-ketoglutarate-dependent dioxygenases, the Jumonji C domain (JMJD) containing histone lysine demethylases (KDM) and the ten eleven translocation (TET) methylcytosine DNA demethylases (reviewed in [21,22]). Modulation of the α-ketoglutarate/succinate ratio is sufficient to regulate histone and DNA methylation that direct pluripotency-associated gene expression [21]. Other products of the tricarboxylic acid (TCA) cycle have less well-explored biology inside the nucleus [22,23] and this especially in form of reactive acyl-CoA compounds [24].

Histone lysine succinylation is a nonenzymatic modification originating from succinyl-CoA [25]. It can be actively removed by SIRT5 deacylase [26]. Histone succinylation sites have been mapped in various organisms, such as yeast, fruitfly, mouse and human cell lines, where they account for 13, 7, 10 and 7 sites, respectively [27,28]. Succinyl-CoA also stimulates HDAC activity *in vitro.* And it has been speculated that such effect might also be exerted by succinylated histones [27].

Fumarate and succinate inhibit the α-ketoglutarate-dependent dioxygenases, thereby affecting DNA replication and stability [29]. Fumarate may also serve as a substrate for succinylation of histone cysteines via nonenzymatic addition of fumarate to the thiol group of cysteines [22,30]. However, this modification has so far not been mapped to chromatin and its effects are not explored.

### Methylglyoxal and 3-deoxyglucosone

Glycation of proteins is a common nonenzymatic covalent modification in diabetes and other hyperglycemic states. Glycation results from the condensation of the aldehyde form of monosaccharides or glycolytic by-products (such as methylglyoxal and 3-deoxyglucosone (3-DG)) with reactive amino acid residues (mainly lysines and arginines) via a nonenzymatic reaction [31]. This reaction was found to happen with methylglyoxal and all core histones, with prevalence of H3 [31]. Glycation of histones causes destabilization of nucleosomes *in vitro* and *in cellulo*, possibly by attaching to critical residues that are serving as intranucleosomal anchors and as binding sites for chromatin readers [32]. It is not clear whether glycation has specific downstream effectors, though it is actively removed from histones by the DJ-1 deglycase enzyme, which reduces disturbing effects of this modification [31].

3-DG is an α-oxoaldehyde, a reactive compound involved in vascular damage in diabetes. As with methylglyoxal, 3-DG reacts with histones, and glycation leads to the generation of advanced glycation end-products (AGEs). *In vitro* studies of 3-DG treatment of H1, H2A and H3 have shown that their secondary structures were altered upon glycation [33]. Histone glycation affects stability and function, as well as is implied in the autoimmune response [34].

### Acetyl-ADP-ribose

ADP-ribosylation reactions can be divided into four groups: mono-ADP-ribosylation, poly-ADP-ribosylation, ADP-ribose cyclization, and formation of *O*-acetyl-ADP-ribose (AAR) [35]. Here, we discuss only AAR and poly-ADP-ribose (PAR, see next section) for their relevance in chromatin biology. These metabolites are derived from NAD^+^ and act as secondary messengers in chromatin organization processes.

AAR is a by-product of histone deacetylation and NAD^+^ hydrolysis, catalyzed by Sirtuin proteins in a wide range of model organisms. It can act as a signaling molecule and substrate for chromatin-modifying enzymes [36], and its level can also be regulated by designated Nudix (nucleoside diphosphate linked to another moiety X) hydrolases [37].

AAR regulates Silent Information Regulator (SIR) complex function in yeast [38]. SIR proteins mediate heterochromatin silencing. Sir2 initiates the formation of silent chromatin by deacetylation of histones, producing AAR. AAR in turn binds to Sir3 complexes and stabilizes the interaction with nucleosomes and oligomerization with other Sir3 proteins, thus provoking formation and spreading of heterochromatin [39]. It was also determined that AAR stays in a stable association with established heterochromatin [40].

In higher eukaryotes, SIRT1, a deacetylase associated with heterochromatin [41] also produces AAR as a by-product of deacetylation. *In vitro*, AAR is specifically recognized by the histone variant macroH2A1.1 via its macrodomain [42,43]. Histone variants substitute for the canonical core histones in specific regions of the genome and under defined conditions. While these generally support nucleosome assembly, the various changes in amino acid composition compared with the canonical histones confer changes in function. It is yet unclear what exactly the function of AAR binding to macroH2A1.1 is [44,45]. But the fact that macroH2A1.1 is enriched in repressive chromatin [46], and that only this splicing variant of macroH2A1 but not macroH2A1.2 strongly binds to AAR [42] suggests a specific role of AAR in heterochromatin formation. Since AAR production by sirtuins is evolutionarily conserved in organisms that do not contain macroH2A, and as not all macroH2As of different organisms seem to bind AAR [47], the existence of other reader proteins of AAR was suggested [48]. AAR has already become an attractive target for designing regulatory protein inhibitors [49,50].

### PAR

PAR is a negatively charged polymer with variable structure of two or more ADP-ribose units that are derived from NAD^+^ [51]. PAR is attached to proteins at different amino acids, including aspartate, glutamate, and lysine residues via enzymes of the PAR polymerases (PARPs) protein family [52,53]. The PARPs add single or multiple ADP-ribose units either connecting the ribose group of one ADP-ribose unit to the adenosine of the adjacent ADP-ribose unit (resulting in linear PAR polymers) or linking the nonadenosine ribose groups from neighboring ADP-ribose units (generating branched PAR) [54]. The levels of PAR are controlled by specific hydrolases, such as PARG.

PAR has many divergent roles in cellular signaling, chromatin maintenance, and stress response (reviewed in [33,52,53]). The polymer regulates nuclear processes via disruption of protein and DNA interactions, scaffolding of protein complexes, and cross-talking with other protein PTMs. Besides roles of PAR in establishing stress granules [55], organization of the nucleolus [56] and mitotic spindle formation [57], particularly its involvement in the DNA damage response is well documented [58].

PARP1, the most studied PARP protein, is recruited to DNA strand-break sites and initiates local PAR synthesis. PAR is either covalently attached to histones H1 and H2A [59] and to other proteins (including PARP itself) [60], or accumulates as a free molecule [35]. PARylation of histones is linked to chromatin decondensation, which in turn allows for local DNA accessibility and promotes DNA repair [61]. PAR also recruits downstream effectors. For example, a chromatin remodeler ALC1 (Amplified in Liver Cancer 1, also known as CHD1L) is activated by binding to PAR via its macrodomain [62,63]. ALC1 promotes nucleosome sliding by interacting with the acidic patch on the nucleosome [64], thus amplifying chromatin decompaction. PAR may recruit other proteins to DNA break sites, such as CHFR, an E3 ubiquitin ligase [65] or condensin I [66], that in turn leads to global chromatin structural changes. It is proposed that PAR may regulate gene expression through the same mechanisms (chromatin structural change and recruitment of effector proteins) in normal conditions [67]. Because of the many roles of PAR and its specialized effector proteins, PAR has been targeted for pharmaceutical development [68,69].

### Uridine diphosphate N-acetylglucosamine

Uridine diphosphate N-acetylglucosamine (UDP-GlcNAc) is among the most abundant high-energy metabolites and it is the final product of the hexosamine biosynthetic pathway—a branch of glycolysis responsible for the production of a key substrate for protein glycosylation. O-GlcNAcylation is a PTM of proteins catalyzed by the O-Linked N-acetylglucosamine (O-GlcNAc) transferase (OGT) and reversed by O-GlcNAcase (OGA) [70].

O-GlcNAcylation of all core histones has been described [71], with O-GlcNAc found on histone threonines and serines [72]. Specific sites that have been mapped include H2AT101, H2BS36, H3S10, H3S28, H3T32, and H4S47. Interestingly, the same sites were earlier described as phosphorylation targets, and this is why O-GlcNAcylation is thought to act as a nutrient-dependent sensor competing with phosphorylation signals and their effector pathways [73,74].

The OGT enzyme forms many functionally distinct complexes with other nuclear proteins, which determine the target location and the outcome of O-GlcNAcylation. For example, OGT is directed to chromatin by the TET DNA demethylase proteins, and many other OGT complexes have been described [75]. Recent findings suggest that O-GlcNAcylation directly regulates a range of chromatin-modifying proteins. Glycation of HDAC4 deacetylase was shown to be involved in improving diabetic complications of heart by preventing phosphorylation of the same site, which in turn is associated with a detrimental outcome [76]. O-GlcNAcylation of several residues on histone methyltransferase EZH2 was shown to differently regulate its stability and chromatin interactions. It was proposed that selective inhibition of this modification may be used to specifically modify EZH2 activity [77].

With only single copies of OGT and OGA enzymes in humans, and many roles of O-GlcNAcylation in the cell nucleus, UDP-GlcNAc metabolism is both an attractive target for modulation of its downstream effect and a vulnerable point involved in several pathologies [75].

### Inositol polyphosphates

The *de novo* myo-inositol biosynthesis pathway converts glucose-6-phosphate into inositol 1,4,5-trisphosphate (IP3). IP3 can be subsequently phosphorylated by inositol kinases yielding several phosphorylated species (e.g. inositol 1,4,5,6 tetrakisphosphate (IP4), inositol 1,3,4,5,6 pentakisphosphate (IP5), inositol 1,2,3,4,5,6 hexakisphosphate (IP6), and 5-diphosphoinositol pentakisphosphate (IP7)) [78,79]. The second cellular source of inositol polyphosphates (IPs) is hydrolysis of membrane phospholipids—a process tightly involved in cellular signal transduction, where IPs act as messenger molecules mediating various processes.

IPs are highly negatively charged and they exert chromatin effects by interacting with specific protein targets. For example, IP6 inhibits nucleosome mobilization by the chromatin remodeling NURF, ISW2, and INO80 complexes. In contrast, IP4 and IP5 stimulate the chromatin remodeling (nucleosome-sliding) activity of SWI/SNF [80]. Besides control of enzymatic activity, the IPs direct the interaction of chromatin remodelers with the transcription factors Pho2 and Pho4, which is involved in regulation of some phosphate-responsive genes and inducing their transcription [81].

The enzymatic activity of class I HDAC1, 2 and 3, which form the catalytic subunits of several large transcriptional repression complexes, has been shown to be regulated by IPs. Initially, IP4 was found to be an intrinsic scaffolding part located at a binding pocket formed at the interface between the HDACs and their cognate corepressors NuRD, CoREST, SMRT, Sin3L/Rpd3L and others [82–87]. It was then demonstrated that IP4 allosterically activates the enzymatic activity of the HDACs which are not functional in its absence and outside the corepressor complexes [88–90]. Interestingly, IP5 and IP6 can substitute for IP4 at least *in vitro* [90]. It is at present not fully clear whether the IPs functionally regulate HDAC activity upon signaling events or serve as constitutive cofactors of the enzyme complexes.

IP7 functions to regulate its target proteins through allosteric interactions or protein pyrophosphorylation. Inositol hexakisphosphate kinase 1 (IP6K1), the enzyme responsible for the synthesis of IP7, associates with chromatin and interacts with the histone lysine demethylase, Jumonji domain containing 2C (JMJD2C). IP6K1 induces JMJD2C dissociation from chromatin and increases H3K9me3 levels, which depend on IP6K1 catalytic activity. Reducing IP6K1 expression levels or IP7 concentration causes a reduction in the levels of the H3K9me3 modification concomitant with an increase in the H3K9ac mark. The molecular mechanisms of this effect are not yet known but this phenomenon shows that there is localized production of IP to control histone methylation [91].

## Chromatin-regulating metabolites originating from lipids

### Cholesterol

Cholesterol possesses a tetracyclic steroid ring. It is synthesized via the mevalonate pathway, in which acetyl-CoA is converted by subsequent reactions into several fundamental end-products, including cholesterol and isoprenoids [92].

Cholesterol influences chromatin structure and stability by directly binding to nucleosomes inducing compaction of long chromatin fibers [93–95]. Docking and molecular dynamics simulations suggested that cholesterol interacts with nucleosomes through six binding sites nearby important interacting regions for nucleosome-binding proteins. Cholesterol is proposed to influence critical packing interactions at the nucleosome core particle and facilitate dewetting (i.e. exposure) of hydrophobic surfaces on the histones, leading to enhanced histone–histone contacts that drive chromatin condensation [96,97].

Further on the notion that cholesterol drives the compaction of nucleosomes, cholesterol was shown to be required for the transcriptional repressor brain acid soluble protein 1 (BASP1)-dependent histone modification and transcriptional repression. As such, BASP1-mediated recruitment of cholesterol might elicit direct effects on the nucleosome that contribute to establishing transcriptionally repressive condensed chromatin at specific genes; yet, further studies are needed to elucidate the exact mechanisms of action [98].

### Phosphoinositides

Phospholipids are a class of lipids that consists of fatty acids modified with phosphate groups, and in the case of the glycerophospholipids species, they contain an additional glycerol that bridges multiple fatty acid chains. A prominent example of glycerophospholipids are phosphoinositides (also referred to as phosphatidylinositol phosphates, PIPs). Multiple PIPs that differ in the phosphorylation positions and levels of the inositol polyalcohol are derived from phosphoinositol. The monophosphates are PI3P, PI4P (also referred to often as PIP) and PI5P; the bisphosphates are PI3,4P_2_, PI3,5P_2_ and PI4,5P_2_ (also often referred to as PIP2), and PI3,4,5P_3_ (also often referred to as PIP3) is the trisphosphate. Overall, PIPs occur in low to very low abundance when compared with other lipids. The different PIPs are well known for their biology at the cell membrane and in intracellular vesicle trafficking. In cell signaling PI4,5P_2_ is hydrolyzed to form the second messengers, diacylglycerol (DAG) and IP3. But a separate biology of PIP has been emerging in the nucleus with their concentrations varying in dependence of cellular state (e.g. cell cycle, stress, transcriptional state etc.) [99,100].

Early studies of lipid analysis of isolated animal chromatin revealed different amounts of (phospho-)lipids associated with hetero- and euchromatin. In addition, higher turnover rates of (phospho-)lipids were found associated with euchromatin [101]. Later it was shown that hydrolysis of nuclear phospholipids via phospholipase C (PLC) changes chromatin structure [102]. Also, it was suggested that phospholipids are associated with the chromosomes during mitosis, whereas during interphase, they dissociate from chromatin, with the exception of heterochromatin [103]. Interestingly, addition of phospholipids to purified nuclei can affect transcription and replication of DNA *in vitro* [104]—an effect that is paralleled by changes in chromatin condensation [105]. Over the last years, several chromatin proteins have been found to bind to and be regulated by PIPs.

The ATPase subunit, named BRG-1, of the ATP-dependent chromatin remodeling complex, BAF (the mammalian ortholog of the yeast SWI/SNF complex) binds PI4,5P_2_. The interaction modulates interaction of the BAF complex with actin and tethering to the nuclear matrix [106]. In T cells, the exogenous addition of PI4,5P_2_ causes BAF to translocate from the soluble to the nuclear-insoluble protein fraction which allows it to participate in nuclear architecture formation [107]. BAF complex that contains PI4,5P_2_ bound BRG-1 is not recruited to active promoters [108].

PI4,5P_2_ has further been found to directly bind to histones and in particular linker histone. This interaction is blocked after phosphorylation of H1 by protein kinase C [109]. In a *Drosophila* transcription system, PI4,5P_2_ binding to H1 counteracted repression of RNA polymerase II basal transcription [109]. More recently, PI4,5P_2_ has been shown to regulate the levels of H3K9me2 at the rDNA promoter by binding to and inhibiting the activity of the histone lysine demethylase PHD finger protein 8 (PHF8), likely resulting in a reduction in rRNA gene transcription [110].

The ING2 (inhibitor of growth protein-2), a component of the transcriptional corepressor Sin3a–HDAC1 complex binds PI5P through a plant homeodomain (PHD) finger domain [111,112]. Overexpression of the type II PI5P 4-kinase β, which decreases the levels of nuclear PI5P, alters ING2 translocation to the nucleus and chromatin association [111]. PI5P binding of ING2 has been implicated in the regulation of acetylation of the p53 tumor suppressor and the apoptotic response to DNA damaging agents [113]. PI5P was further demonstrated to be required for the association of ING2 with target gene promoters leading to their transcriptional repression [114]. Other PHD domain containing factors (e.g. ING1b, ING3, ACF, Rag2, PHF1) were also shown to bind PI5P albeit with unknown functional consequence [112]. In contrast, binding of PI5P to the PHD of ATX1, a plant H3K4 trimethyltransferase, causes the enzyme to detach from promoters and translocate from the nucleus to the cytosol [115]. Further, the PHD finger of TAF3 was shown to be affected by interaction with PI5P in its association with the H3K4me3 chromatin mark by a so far unknown allosteric mechanism. This control of a basal transcription factor seems to be involved in regulating gene transcription in myoblast differentiation [116].

Another chromatin protein that has been shown to be functionally regulated by PI5P is UHRF1 (ubiquitin-like with PHD and RING finger domains 1) that plays an important role in reading and maintaining epigenetic states [117]. UHRF1 contains multiple domains for recognizing chromatin modifications and in particular a tandem tudor domain (TTD) that binds H3K9me3 and a PHD domain that binds the unmodified N-terminus of H3. PI5P works as an allosteric regulator of UHRF1 chromatin interaction by inducing conformational rearrangement of the protein thereby establishing a synergistic binding function of the TTD and PHD domains [118,119]. The fact that multiple chromatin proteins with PHD domains that are known to bind methyl-lysines have been found to interact with and be regulated by PI5P raises the possibility of a more general cross-talk between such reader domains and the phosphoinositides. Such notion is supported by a binding screen conducted with 32 different PHD finger sequences that identified 17 of these as interactors of PI5P and other PIPs [116].

Further illustrating the diverse chromatin biology of nuclear PIPs is the association of nucleophosmin, a factor that besides other functions, serves as a histone chaperone, with PI3,4,5P_3_ that was linked to apoptosis [120]. Accumulation of PI3,4,5P_3_ was also found at sites of DNA damage and required for local recruitment of the ATR kinase [121]. The HDAC corepressor components SAP30 and SAP30L are negatively regulated in their association with DNA by different PIPs [122]. Diverse transcription factors that work in dependence of histone-modifying enzymes were shown to be functionally regulated in their chromatin recruitment by PI5P, PI3,5P_2_, PI4,5P_2_, and/or PI3,4,5P_3_ [123]. Different biochemical screens have (besides other proteins) identified additional chromatin factors interacting with PI4,5P_2_ and PI5P albeit with so far unclear functional implications [116,124].

### Sphingolipids

Sphingolipids are a class of lipids containing a single fatty acid chain and amino alcohol group. An example of a nuclear sphingolipid that is involved in chromatin modification is sphingosine. Sphingosine-1-phosphate (S1P) has been found to regulate histone acetylation. Sphingosine kinase 2 (SphK2) which generates S1P, was found to be associated with histone H3 [125]. SphK2 enhanced local histone H3 acetylation via the direct binding of S1P to HDAC1 and HDAC2. S1P specifically binds to the active sites of HDAC1 and HDAC2 and inhibits their enzymatic activities, thereby preventing the removal of acetyl groups from lysine residues within histone tails and leading to increased histone acetylation. Thus, HDACs are direct intracellular targets of S1P and link nuclear S1P to epigenetic regulation of gene expression [126]. In fact, S1P treatment has been reported to ameliorate the cardiac hypertrophic response, which may be partly mediated by the suppression of HDAC2 activity. Therefore, S1P may be considered a potential therapy for the treatment of heart diseases caused by cardiac hypertrophy [127].

### Palmitic acid

Palmitic acid is a major saturated fatty acid of 16 carbons (C16:0). S-palmitoylation and O-palmitoylation describe the addition of palmitic acid to protein cysteine and serine residues, respectively [128]. Thiol side chains of exposed cysteines can attack electrophilic species, including molecules with carboxylic acid substituents, such as palmitic acid [129]. The resulting thioester bond is energetically labile, allowing for reversibility. All H3 variant proteins but CENP-A share a conserved cysteine residue (at position 110 for human H3) as a primary site for S-palmitoylation [130].

O-palmitoylation occurs at histone serine residues whereby the serine hydroxyl group forms an oxygen ester bond with palmitate, that is a more stable bond compared with the cysteine thioester [131]. O-palmitoylation has been detected on H4S47 but not the only other serine residue at position 1, suggesting a specific mode of modification [132]. Indeed, acyl-CoA:lysophosphatidylcholine acyltransferase (Lpcat1) has been shown to colocalize with chromatin under specific conditions. Lpcat1 binds to histone H4 and catalyzes histone H4 palmitoylation at S47 [132]. Mutation of H4S47 reduces H4 O-palmitoylation levels and is concomitant with substantially decreased RNA synthesis. While this suggests a role of H4 O-palmitoylation in transcriptional repression, a more direct connection to regulation of gene expression is yet to be found [132].

### 4-Oxo-2-nonenal

Lipids can be peroxidized to yield highly reactive, electrophilic α,β-unsaturated aldehydes, such as 4-oxo-2-nonenal (4-ONE) that are found to chemically modify a number of proteins and DNA [133]. Lipid peroxidation can be described generally as a process under which oxidants, such as free radicals, attack lipids containing carbon–carbon double bonds. 4-ONE have been shown to form adducts on all core histones *in vitro*, attaching to lysine residues via ketoamide adduction or attaching to histidines residues via Michael addition. Under oxidative stress, the adduct on histones disrupts the interaction with DNA, thereby preventing the assembly of nucleosomes [134].

Because the 4-ONyl modification is similar to fatty acyl groups, sirtuins and particular SIRT2 were suggested to hydrolyze histone 4-ONyl lysine adducts. This action mitigates the negative impact of this modification, namely the inhibition of nucleosome assembly, caused by oxidative stress [135].

## Chromatin-regulating metabolites originating from amino acids

### Glutarate

Glutarate can be derived from the metabolism of the essential amino acids lysine and tryptophan. It can form reactive glutaryl-CoA, and become a donor for glutarylation of proteins [136]. GCN5 (also referred to as KAT2A), which was originally described as a histone acetyltransferase, has been shown to mediate histone glutarylation [137,138]. At present, it is unclear whether GCN5 mediates all histone glutarylation, whether other enzymes for this modification exist or whether some sites are targets of nonenzymatic modification. So far, 27 glutarylation sites on histone lysines in HeLa cells have been mapped [137]. Glutarylated histones are enriched at transcriptional start sites and associated with permissive transcription. By introducing a negative charge, glutarylation is proposed to cause opening of chromatin similar to other acylation modifications and to destabilize the H2A/H2B and H3/H4 interactions within the nucleosome *in vitro* [137]. Since histone glutarylation is generally far less abundant than histone acetylation but appears to mediate similar effects, its independent chromatin signaling needs to be further investigated. Evidence for a physiological role separate from histone acetylation might come from the cellular study of a mimicking modification—H4K91 mutated to glutamic acid, bearing a negative charge like glutaryl itself. It was demonstrated that this substitution has an effect on cell proliferation and DNA damage response in yeast, as well as causing deregulation of a large set of genes [137]. SIRT5 and/or SIRT7 have been suggested to function as deglutarylation enzymes [136,137].

### Homocysteine thiolactone

Homocysteine is an intermediate metabolite in the cysteine and methionine metabolism. S-adenosylhomocysteine (SAH) is the reaction product of SAM after donating an activated methyl-group for methylation reactions. Homocysteine is not proteinogenic, however it can be recognized by methionyl-tRNA and transformed into a cyclic thioester homocysteine thiolactone [139]. In one study, the expression level of genes involved in chromatin organization was altered upon treatment with homocysteine thiolactone [140], making a correlative link between this molecule and chromatin. One of the mechanisms for this link may be a direct histone modification, as homocysteine thiolactone reacts with histone lysines nonenzymatically forming a covalent bond [141]. This modification is particularly interesting in metabolic disorders and hyperhomocysteinemia, as homocysteinylation is regulated by intracellular levels of its precursor. A total of 39 homocysteinylation sites on all of the four core histones were identified in human embryonic brain cells and a possible cross-talk with other lysine modifications, such as acetylation and methylation, has been suggested [141]. H3K79Hcy is involved in fetal neuronal development via down-regulation of *Smarca4*, *Cecr2*, and *Dnmt3b*, genes that are playing critical roles in neural tube closure [142].

### Glutathione

Glutathione (GSH) is a tripeptide that consists of cysteine, glutamate, and glycine. This molecule is mainly known as a redox agent and buffer that prevents damage to cellular components caused by reactive oxygen species. However, there are also links between GSH metabolism and chromatin function on different levels [143,144]. Biosynthesis of GSH competes for substrate with methionine metabolism and SAM/SAH balance, putting it in a relation to DNA and histone methylation processes [143]. But GSH also has more direct roles in chromatin regulation, particularly it is a key molecule that aids rapid chromatin decondensation in sperm nuclei [145].

Most nuclear functions of GSH are associated with its free form [146], however GSH can be also covalently bound to the cysteine residues of H3 via disulfide linkage [143,147]. This attachment seems to happen nonenzymatically. *In vitro* studies showed that addition of GSH destabilizes the nucleosome and leads to an open chromatin state, but the exact functional implications of this modification are not known. The spread of this modification increases during proliferation and decreases with age [143].

### Monoamines (serotonin, dopamine)

Dopamine is a product of the phenylalanine and tyrosine metabolism, whereas serotonin is derived from tryptophan. In addition to their well-known physiological roles as neurotransmitters and neuromodulators, monoamines have also been described in direct chromatin regulation. The extravesicular monoamines can be covalently attached to histones by the transglutaminase (Tgm2) enzyme. Serotonin was found on the H3Q5 residue [148], and this modification was also observed in conjunction with the activating mark H3K4me3. H3Q5 serotonylation has been generally correlated with a permissive transcription state and might function via facilitating binding of the TFIID basal transcription factor to H3K4me3. Histone serotonylation was found to be enriched at genes related to axon guidance and neuronal development [148].

Dopamine is another monoamine that can be used to modify histones, it is attached to H3Q5 by the same mechanism as serotonin [149]. H3Q5 dopaminylation has been studied in a particular context of drug-abuse behavior and cocaine addiction. In the brain region involved in reward behavior changes where dopamine is synthesized, H3Q5 dopaminylation was reduced in samples derived from overdosed cocaine users. In a rat model, the modification is gradually re-established after a prolonged time since the last cocaine administration. Artificially reducing H3Q5 dopaminylation in rats resulted in absence of drug-seeking behavior during withdrawal. The findings imply that this histone monoamine modification has a prolonged effect on cell physiology enacted via disrupting gene expression patterns [149].

### Polyamines (spermine, spermidine)

Polyamines are formed by enzymatic decarboxylation of the amino acids ornithine or arginine. The most common polyamines are spermidine, spermine, and their biosynthetic precursor putrescine. Though the primary role of polyamines seems to be in RNA translation in the cytoplasm [150], they are also imported into the nucleus and involved in several processes, including chromatin conformation maintenance, DNA replication, and gene expression [151]. There are several controversial observations (which might be explained by the differences in experimental systems) about a direct action of polyamines on chromatin components (for a detailed review, see [152]). Some of the peculiar examples are induction of DNA conformational change from B-DNA to Z-DNA [153], *in vitro* chromatin condensation at the nucleosome level, and modulating of expression and activity of chromatin-modifying enzymes (HAT, HDAC, LSD1) upon polyamines administration or depletion. Interestingly, polyamines are themselves subject to modification by acetylation. This regulates their chromatin association [154] and also cross-talk to histone acetylation [155]. To this point, the findings are largely correlational and do not yet provide a detailed mechanistic insight into the nuclear functions of polyamines.

Despite the absence of a clear understanding of polyamines function on chromatin, polyamine-based anticancer drugs are being developed. Polyamine analogs, such as polyaminohydroxamic acids (PAHAs) and polyaminobenzamides (PABAs), demonstrated potent inhibition of HDACs, re-expression of p21 and significant inhibition of tumor growth [156].

## Origin of metabolites regulating chromatin biology

With the small size, the metabolites we have discussed can likely freely diffuse in and out of the nucleus though its pores. While this has been postulated to establish a connection between the cytoplasmic and nuclear state of a cell [157,158], it is emerging that many enzymes controlling the concentration of metabolites regulating chromatin biology localize to the nucleus under specific cellular conditions or have specific isoforms that are constitutively found in the vicinity of chromatin [11,159–161].

This phenomenon is now well documented for enzymes metabolizing the common and well-studied substrates and cofactors of chromatin modification. For example, the acetyl-CoA synthesizing enzymes acyl-CoA synthetase short-chain family member 2 (ACSS2), ATP-citrate lyase (ACLY), and the pyruvate dehydrogenase complex (PDC) are all found nuclear besides their cytoplasmic or mitochondrial primary localization [159,160]. Similarly, methionine adenosyltransferase 2A (MAT2A) that converts methionine into SAM is found not only in the cytoplasm but also in the nucleus [162]. Of relevance for the consideration of unconventional metabolites in chromatin regulation, different enzymes of carbohydrate metabolism including the glycolytic enzymes pyruvate kinase M2 (PKM2), 6-phosphofructo-2-kinase/fructose-2,6-bisphosphatase 4 (PFKFB4), fructose-1,6-bisphosphatase 1 (FBP1), glyceraldehyde-3-phosphate dehydrogenase (GAPDH), and triose phosphate isomerase 1 (TPI1) were described with nuclear localization under specific conditions [11,163]. Further, several enzymes of the TCA cycle such as α-ketoglutarate dehydrogenase (α-KGDH) and fumarase (lactate dehydrogenase, LDH, [164]) have a nuclear appearance. Indeed, all mitochondrial enzymes executing the different steps of converting pyruvate into α-ketoglutarate were found to transiently operate in the nucleus of the developing zygote [165]. The pathways of synthesis and salvage of NAD^+^ are also compartmentalizing to the cell nucleus [166,167]. This seems particularly interesting in light that NAD^+^ itself is consumed in poly-ADP-ribosylation or production of acetyl-ADP-ribose instead of changing its oxidation state as in cytoplasmic energy conversion. Also, several enzymes of inositolphosphate metabolisms such as phospholipase C and different inositol phosphate kinases are suggested to have a nuclear life based on screens conducted in yeast [168], and several of these seem to shuttle in and out of the nucleus [169].

For the chromatin-regulating metabolites linked to lipids, it is interesting to notice that two main enzymes involved in lipid biosynthesis, acetyl-CoA carboxylase 1 (ACC1) that catalyzes the rate-limiting step of lipid biosynthesis, the conversion of acetyl-CoA into malonyl-CoA and fatty acid synthase (FASN) have been described to show oscillations between cytoplasmic and nuclear localization [170]. Very well documented are specific isoforms of different kinases and phosphatases that interconvert the different phosphoinositides and which are found exclusively in the cytoplasm or in the nucleus [171,172]. Other enzymes of PIP metabolism change their subcellular localization upon defined stimuli, which has been linked to dynamic changes of the nuclear PIP pool [123,173,174].

Besides a general nuclear presence, it is more and more emerging that different metabolic enzymes might be recruited to specific chromatin regions, for example via interaction with transcription factors [175–180]. This has been proposed to modulate the local metabolite concentration of genomic microdomains, thereby establishing local hubs of metabolite production and chromatin regulation [181]. The recent discovery of phase separated systems in the cell nucleus that seem to establish localized hubs of concentrated enzymes and regulation further supports such idea [160].

## Perspective

Clear linkage among cellular state, metabolism, chromatin status, and epigenetics has been established over the last years [182–185]. While several central metabolites have been extensively studied in this context and in different model systems [4–13], we deduce from our discussion of less common metabolites and their effects on chromatin signaling that the full extent of this connection cannot yet be comprehended.

First, there is a plethora of options where metabolites that might affect chromatin could originate (see Figure 2). Besides primary and secondary metabolism, this includes molecules that are ‘by-products’ of classical biosynthesis and degradation pathways. At present, there are, however, only limited experimental schemes that can identify and characterize metabolites with chromatin function. The most developed approach is the mass spectrometry characterization of covalent histone modifications [186–188]. Not surprisingly, the majority of uncommon metabolites that we discussed here form nonenzymatic or enzymatic histone PTMs. Identifying metabolites with direct chromatin effects or modulating the biology of chromatin regulators (Figure 1), however, is far less straightforward. In many cases, the chromatin biology of metabolites in these categories was discovered by accident or serendipity. For example, the essential role of IP4 in activation of class I HDACs only became apparent after purification and crystallization of corepressor complexes isolated from mammalian cell lines [82]. Identifying the chromatin functions of PIPs was based on educated guesses [111] and/or cumbersome purification schemes derived from activity assays [118]. We believe that many more metabolites with roles in chromatin biology will be discovered once systematic approaches for their identification and in different functional context become available.

Second, the interfaces metabolites can have with chromatin systems are plenty and there are multiple levels where these might exert chromatin regulatory function (see Figure 1). Our discussion of less common metabolites in chromatin biology revealed that examples for most of the (theoretical) options for molecular working modes do exist. In this context, it needs to be acknowledged that not all metabolites we looked at have been studied to the same degree (see Table 1). The fact that for different metabolites such as PAR, IP, and PIP multiple modes and functions in chromatin regulation have been identified, makes us, nonetheless, hypothesize that many more links between already known as well as still to be described metabolites with roles in chromatin biology await discovery. This will, however, require new and additional experimental procedures. At present, the functional characterization of metabolites in question is mostly driven by *in vitro* characterization of chromatin effects and modulation of binding or enzymatic activities. The *in cellulo* and *in vivo* analysis of the biology of chromatin-regulating metabolites requires approaches that enable the perturbation, control, and quantification of cellular (better nuclear) levels of the molecules in question. Currently, this is limited to exogenous administration of metabolites or their precursors and/or manipulation of the enzyme system controlling their (sub-)cellular levels. Both approaches are, however, fully indirect which makes drawing solid conclusions challenging.

**Table 1:** Chromatin biology of unconventional metabolites

Type	Class	Metabolite	Target	Mechanism of function	Proposed biological effect	Level of evidence
carbohydrates		lactate	core histones	enzymatically catalyzed addition	chromatin decompaction; transcriptional activation; recruitment of downstream effectors	several *in vitro*, *in cellulo*, and *in vivo* studies in tissues and cells undergoing fluctuations of lactate (hypoxia, tumor, immune cells, stem cells)
			histone deacetylases	inhibition	chromatin decompaction; transcriptional activation	few studies using cell lines
	intermediates of the TCA cycle	succinyl-CoA	core histones	nonenzymatic covalent attachment	destabilization of the nucleosome; affects viability in yeast	mapped in different experimental systems; few *in vivo* studies (mutagenesis of succinylated lysine residues in yeast)
			histone deacetylases	activation	regulation of chromatin acetylation levels	few *in vitro* studies
		succinate and fumarate	DNA and histone demethylases	inhibition	chromatin replication and stability	multiple evidence from yeast genetics; different *in vitro* studies
		methylglyoxal; 3-deoxyglucosone	core histones	nonenzymatic covalent attachment	destabilization of the nucleosome leading to an open chromatin state	few studies looking at cell lines cultured on enriched media; different *in vitro* studies
	ADP-ribosylation	acetyl-ADP-ribose (AAR)	Sir3	binding to chromatin-modifying proteins	formation and spreading of heterochromatin	multiple *in vivo* studies in yeast; *in vitro* binding experiments
			macroH2A1.1	binding to histone variant	not yet known	several studies using human cell lines; structural evidence
		poly-ADP-ribose (PAR)	core histones	enzymatically catalyzed addition	chromatin decompaction; recruitment of downstream effectors	multiple studies in different *in vivo*, *in cellulo*, and *in vitro* systems including pathological conditions
		UDP-GlcNAc	core histones	enzymatically catalyzed addition	cross-talk with other histone marks	multiple studies in different *in vivo*, *in cellulo*, and *in vitro* systems
			readers erasers	enzymatically catalyzed addition	stability; chromatin interaction	multiple studies in different *in vivo*, *in cellulo*, and *in vitro* systems
	inositol polyphosphates (IPs)	IP4	class I HDAC	allosteric activation	chromatin and genome control	multiple studies in different *in vivo*, *in cellulo*, and *in vitro* systems; structural evidence
		IP4, IP5, IP6: chromatin remodelers	chromatin remodelers	regulation of activity; interaction with transcription factors	transcriptional regulation	several studies in yeast
		IP7	JMJD2C (histone demethylase)	dissociation from chromatin	ratio of repressive H3K9me3 and activating H3K9ac histone marks	several studies using different cell lines
lipids		cholesterol	chromatin	direct binding	chromatin compaction; transcriptional repression	several *in vitro* studies; *in silico* prediction
			BASP-1	interaction	chromatin compaction; transcriptional repression	few studies in cell lines
	phosphoinositides (PIPs)	PI5P5	UHRF1 (reader)	allosteric activation	interaction with H3K9me3	few *in vitro* studies; few studies in cell lines
			ATX1 (histone methylase)	nuclear localization	transcriptional regulation	few *in vitro* studies; few studies in plants
			TAF3 (basal transcription factor)	control of activity	interaction with H3K4me3; transcriptional regulation	few *in vitro* studies; few studies in plants
			ING2 (reader)	nuclear localization; chromatin association	acetylation of p53; apoptotic response to DNA damage; transcriptional regulation	few *in vitro* studies; few studies in plants
		PI4,5P_2_	BRG1 (chromatin remodeler)	binding to chromatin	transcriptional regulation	few *in vitro* studies; few studies in cell lines
			H1 (linker histone)	direct binding	transcriptional regulation	few *in vitro* studies; few studies in cell lines
			PHF8 (histone demethylase)	regulation of activity	rRNA gene transcription	few *in vitro* studies; few studies in cell lines
		PI3,4,5P_3_	nucleophosmin (histone chaperone)	not yet known	apoptosis	few studies in cell lines
		diverse PIPs	SAP 30, SAP30L (HDAC corepressor components	not yet known	not yet known	few *in vitro* studies; few studies in cell lines
			different transcription factors	chromatin recruitment	transcriptional regulation	few *in vitro* studies; few studies in cell lines
		sphingosine-1- phosphate	HDAC1; HDAC2	inhibition	transcriptional activation	several *in vitro* studies and in different cell lines
		palmitic acid	histones H3 and H4	S-palmitoylation: nonenzymatic covalent attachment	not yet known	few indirect observations in cell lines
				O-palmitoylation: enzymatically catalyzed addition	decreased RNA synthesis, suggesting transcriptional repression	few indirect observations in cell lines
		4-oxo-2-nonenal	core histones	nonenzymatic covalent attachment	inhibition of nucleosome assembly	few studies *in vitro* and in cell lines; relevant in oxidative stress
amino acids		glutarate	core histones	enzymatically catalyzed addition	chromatin decompaction; transcriptional regulation	several studies *in vitro* and in cell lines; relevant in metabolic disorder
		homocysteine thiolactone	core histones	nonenzymatic covalent attachment	not yet known; cross-talk with other histone marks?	few studies in cell lines; relevant in metabolic disorder
		glutathione	histone H3	nonenzymatic covalent attachment	chromatin decompaction; destabilization of the nucleosome leading to an open chromatin state	few studies in cell lines
	monoamines	serotonin, dopamine	histone H3	enzymatically catalyzed addition	transcriptionally permissive chromatin state	few *in vivo* studies (neuronal cells) and in cell lines
	polyamines	spermine, spermidine	chromatin	direct binding	various effects not fully understood	multiple studies in different *in vivo*, *in cellulo* and *in vitro* systems

Third, we project that different metabolites and their multiple modes of chromatin function will open future avenues for intervention with important pathways of genome regulation. On one hand, the levels of chromatin-regulating metabolites might be changed in different disease states. Determining and controlling their levels could provide interesting avenues for diagnosis and therapy. On the other hand, the regulatory mode metabolites exert on chromatin modifiers and in particular via allosteric regulation might enable novel approaches for specifically interfering with histone-modifying enzymes, chromatin remodelers, and histone modification-binding proteins.

There are clearly many things to be discovered about the interplay of metabolites and chromatin, and we are looking forward to new findings in this exciting research area in the near future.

## Abbreviations

AAR: *O*-acetyl-ADP-ribose
ALC1: amplified in liver cancer 1
ATP: adenosine triphosphate
BASP1: brain acid soluble protein 1
CENP_A: centromere protein A
EZH2: enhancer of zeste homolog 2
GSH: glutathione
HDAC: histone deacetylase
H3K9me3: histone H3 lysine 9 trimethylation
ING2: inhibitor of growth protein-2
IP: inositol polyphosphate
IP3: inositol 1,4,5-trisphosphate
IP4: inositol 1,4,5,6 tetrakisphosphate
IP5: inositol 1,3,4,5,6 pentakisphosphate
IP6: inositol 1,2,3,4,5,6 hexakisphosphate
IP6K1: inositol hexakisphosphate kinase 1
IP7: 5-diphosphoinositol pentakisphosphate
JMJD2C: Jumonji domain containing 2C
Lpcat1: lysophosphatidylcholine acyltransferase
NAD^+^: nicotinamide adenine dinucleotide
OGA: O-GlcNAcase
OGT: O-GlcNAc transferase
O-GlcNAc: O-linked N-acetylglucosamine
PAR: poly-ADP-ribose
PARP: PAR polymerase
PHD: plant homeodomain
PHF8: PHD finger protein 8
PIP: phosphatidylinositol phosphate
PTM: post-translational modification
SAH: S-adenosylhomocysteine
SAM: S-adenosylmethionine
SIR: silent information regulator
SphK2: sphingosine kinase 2
SWI/SNF: switch/sucrose non-fermentable
S1P: sphingosine-1-phosphate
TCA: tricarboxylic acid
TET: ten eleven translocation
TTD: tandem tudor domain
UDP-GlcNAc: uridine diphosphate N-acetylglucosamine
UHRF1: ubiquitin-like with PHD and RING finger domains 1
3-DG: 3-deoxyglucosone
4-ONE: 4-oxo-2-nonenal
